# Identifying Irregular Potatoes Using Hausdorff Distance and Intersection over Union

**DOI:** 10.3390/s22155740

**Published:** 2022-07-31

**Authors:** Yongbo Yu, Hong Jiang, Xiangfeng Zhang, Yutong Chen

**Affiliations:** College of Mechanical Engineering, Xinjiang University, Urumqi 830017, China; yuybo@stu.xju.edu.cn (Y.Y.); xjuzxf@xju.edu.cn (X.Z.); chenyut@stu.xju.edu.cn (Y.C.)

**Keywords:** irregular potatoes, ellipse fitting, Hausdorff distance, least squares, machine vision

## Abstract

Further processing and the added value of potatoes are limited by irregular potatoes. An ellipse-fitting-based Hausdorff distance and intersection over union (IoU) method for identifying irregular potatoes is proposed to solve the problem. First, the acquired potato image is resized, translated, segmented, and filtered to obtain the potato contour information. Secondly, a least-squares fitting method fits the extracted contour to an ellipse. Then, the similarity between the irregular potato contour and the fitted ellipse is characterized using the perimeter ratio, area ratio, Hausdorff distance, and IoU. Next, the characterization ability of the four features is analyzed, and an identification standard of irregular potatoes is established. Finally, we discuss the algorithm’s shortcomings in this paper and draw the advantages of the algorithm by comparison. The experimental results showed that the characterization ability of perimeter ratio and area ratio was inferior to that of Hausdorff distance and IoU, and using Hausdorff distance and IoU as feature parameters can effectively identify irregular potatoes. Using Hausdorff distance separately as a feature parameter, the algorithm achieved excellent performance, with precision, recall, and F1 scores reaching 0.9423, 0.98, and 0.9608, respectively. Using IoU separately as a feature parameter, the algorithm achieved a higher overall recognition rate, with precision, recall, and F1 scores of 1, 0.96, and 0.9796, respectively. Compared with existing studies, the proposed algorithm identifies irregular potatoes using only one feature, avoiding the complexity of high-dimensional features and significantly reducing the computing effort. Moreover, simple threshold segmentation does not require data training and saves algorithm execution time.

## 1. Introduction

The potato is the fourth largest food crop in the world [1] and is grown in more than 150 countries and regions. As of 2020, potatoes will be grown on about 19.59 million hectares, with Asia and Europe as the central potato-growing regions. The potato provides nutrition for more than one billion people worldwide and is an essential guarantor of food security [2]. In China, the potato-planting area is stable, at more than 80 million mu, with an annual output of 100 million tons of fresh potatoes [3]. Shape is one of the most important indicators of the external quality of potatoes [4]. Regularly shaped potatoes are preferred by consumers, have stronger sales appeal, and play an important role in processing chips and fries [5]. When part of the potato grows under hot and dry conditions, the shape of the potato is changed, resulting in irregular potatoes. Irregular potatoes seriously affect pricing, significantly reducing the economic benefits of the crop [6]. With the increasing human demand for direct potato consumption and food processing, the market demand for regular potatoes is also growing [7]. However, most irregular potatoes currently go directly to the market without being removed, which seriously affects consumers’ desire to buy, as well as the added value of potatoes [8]. Therefore, accurately identifying irregular potatoes can guarantee that they are appropriately discarded to prevent them from reaching the market, which is extremely important for the food production chain [9].

Currently, the primary methods used for shape inspection are manual expert inspection and machine vision inspection [10]. The traditional techniques for detection of irregular potatoes include subjective determination by the naked eye of a grading expert, which lacks a unified identification standard [11,12]. Crop shape detection based on machine vision mainly uses image processing, pattern recognition, machine learning, and deep learning technologies to analyze crop images, which can effectively identify the shapes of crops [13]. Few studies have been conducted focusing on identification of irregular potatoes, and most have focused on potato shape grading based on traditional image processing methods [14,15]. Wang et al. [16] extracted potatoes’ contour area and external rectangle and used principal component analysis (PCA) to analyze the relationship between image feature parameters and potato shape. Zheng et al. [17] used the normalized radius sequence method to inspect irregular potatoes. Deng et al. [18] estimated potato shape by calculating the ratio of potato contour length and the equivalent ellipse perimeter. Zhou et al. [19] determined potato shape grading standards based on the ratio of the maximum transverse diameter to the maximum longitudinal diameter of the potato. Lopez-Juarez [20] proposed a boundary object function to detect the shape of potatoes. Tao et al. [21] proposed a shape separation method based on Fourier transform for automatic inspection of potato shapes. Cui et al. [22] proposed a potato shape recognition method based on Fourier descriptors of moment features of boundary points, which classifies potatoes shapes ellipse, circle, or irregular. Kong et al. [23] used the six invariant moments of the top view image of potatoes as grading features and achieved grading using a BP neural network. ElMasry et al. [4] extracted two shape features and four Fourier shape descriptors based on linear discriminant analysis (LDA) to efficiently identify the shapes of potatoes. Aziz and Abbaspour-Gilandeh [24] proposed a method combining geometric parameters and Fourier descriptors to detect irregular potatoes, using PCA to select the seven most prominent features to complete the grading. Xu and Zhao [25] proposed a potato shape-grading method combining principal component analysis and a support vector machine (PCA-SVM) algorithm to sort potato shapes with 11-dimensional feature vectors. Shen et al. [26] extracted geometric characteristics, image wavelet moment, and fractal boundary dimensions as feature parameters and completed potato shape identification using a support vector machine (SVM) with an accuracy of 88.89%. Deep learning has developed rapidly in recent years and has been widely used in industry and agriculture [27]. Deep learning has been successfully applied in agriculture [28] and, recently, for potato defect detection. Marino et al. [29] proposed a weakly supervised learning method to classify six defects in potatoes, using convolutional neural networks (CNN) for the classification task. Oppenheim et al. [30] used a deep convolutional neural network trained on a potato defect dataset to classify potato tubers into five categories. Zhang et al. [31] used the improved YoloV4 model to detect potato defects and achieved an average accuracy of 91.4% for potato defect identification.

Although these methods have achieved good inspection and grading results, they are subject to certain limitations. For example, shape classification based on simple geometric features cannot cope with the complex shape of irregular potatoes. Fourier shape descriptors work well for round and oval potatoes but not for complicated irregular potatoes and are susceptible to noise and local information interference, meaning they lack robustness. Invariant moments increase the computational effort, making them unsuitable for applications requiring real-time performance. Manually designed features are high in accuracy with respect to training sets but need to be redesigned if new irregular features appear. More importantly, existing high-accuracy shape detection methods use more than one feature, leading to a high dimensionality of the feature parameters and increasing the grading effort. Deep learning approaches require massive datasets and tedious and time-consuming data-labeling and training efforts. In practical industrial applications, deep learning also demands advanced hardware facilities, with considerable associated costs.

With the maturity and perfection of image processing and machine vision technology, as well as the development needs of precision agriculture, countries have begun to study the use of computer vision technology for the grading and inspection of agricultural products [32]. Machine vision inspection has the advantages of economy, objectivity, and high index, which overcome the disadvantages of high labor costs, low efficiency, vague grading standards, and subjectivity of manual expert inspection. It has become a hot research topic in crop shape inspection [33]. However, existing methods use high-dimensional feature parameters to accurately detect the complex variable shapes of irregular potatoes. It is still challenging to effectively and accurately describe the shape of potatoes using a minimal number of features [34].

With this problem in mind, a method of irregular potato identification based on Hausdorff distance and IoU is proposed to achieve accurate identification of irregular potatoes with a minimum number of features. The contributions of this paper are as follows:

1. Construction of a dataset of potatoes: after resizing, translation, graying, segmentation, morphological filtering, and median filtering, a canny edge-detection operator is used to extract potato contours;
2. Using the least-squares method to fit the potato contour to an ellipse, the perimeter ratio, area ratio, Hausdorff distance, and IoU feature parameters are extracted;
3. We analyze the characterization ability of perimeter ratio, area ratio, Hausdorff distance, and IoU. Experimental validation showed that the characterization ability of perimeter ratio and area ratio is inferior to that of Hausdorff distance and IoU. Therefore, Hausdorff distance and IoU are suitable feature parameters for identifying irregular potatoes;
4. A suitable threshold value is determined to identify irregular potatoes. Furthermore, standards for identifying irregular potatoes are established. The experimental results show that the two proposed features have excellent recognition ability, with a maximum F1 score of 0.9796.

The remainder of this paper is organized as follows. In Section 2, we introduce the experimental sample and the vision acquisition device and details the algorithm’s overall flow. In Section 3, we analyze the characterization ability of the four features, establish a standard for identifying irregular potatoes, and present the experimental results. In Section 4, we discuss the problems encountered in execution of the algorithm and draw its advantages. In Section 5, we present our conclusions.

## 2. Materials and Methods

### 2.1. Potato Samples

**Figure 1:**
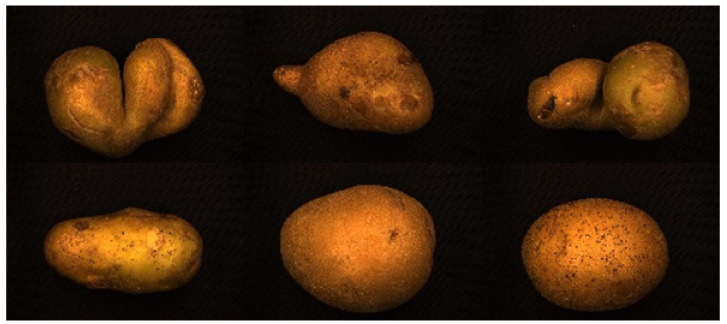
Various regular and irregular potatoes.

There is a wide variety of potatoes, with more than a thousand varieties available worldwide. According to external shape and color, potatoes can be divided into seven broad categories [35], as shown in Appendix A. XiSen 6 potatoes, native to Urumqi, Xinjiang, China, are used as the experimental material in this experiment. They are yellowish-brown in appearance, with three types of shapes: round-like, oval, and irregular. The test sample consisted of 273 potatoes, including 75 irregular potatoes. According to the definition of irregular potato in national standard [36] GB/T 31784-2015 “Code of practice for grading and inspecting of commercial potatoes“ (irregularity: not conforming to the original morphological characteristics of the tubers of the variety), the experimental potato samples are manually classified by experienced potato grading experts into two categories: regular potatoes and irregular potatoes. Regular potatoes and various irregular potatoes are shown in Figure 1.

### 2.2. Vision Acquisition System

**Figure 2:**
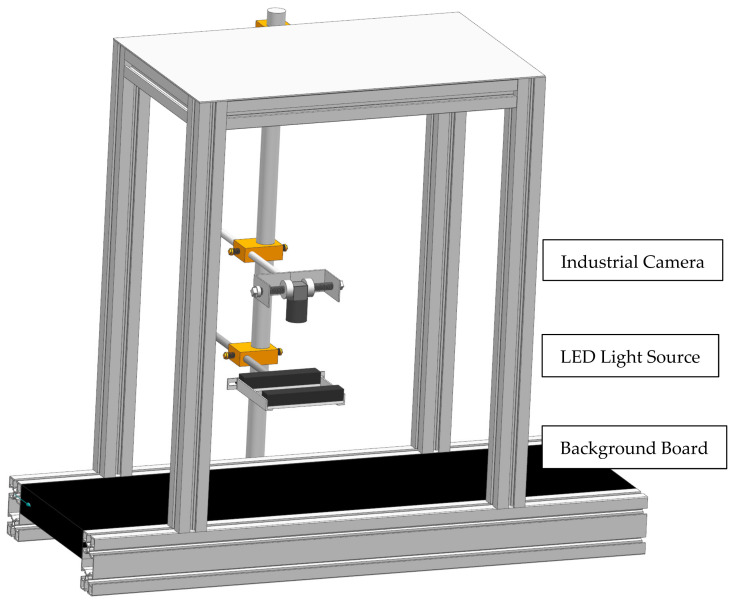
Vision acquisition system.

As shown in Figure 2, the vision acquisition system consists of a shooting background board, an industrial camera, an LED strip light source, and an upper host computer.

The principal parameters of the vision acquisition system are shown in Table 1. The industrial camera is an MV-CA060-10GC from HIKROBOT, China, with Sony’s IMX178 CMOS chip and fast real-time data transmission to the host computer via a Gigabit LAN port, a maximum frame rate up to 17 fps, 6 million pixels, and image resolution of 3072 × 2048. The camera lens faces downward vertically to photograph potatoes, with a reasonable working distance of 300 mm. The light source is an LED strip, shining vertically onto the potato surface, with a color temperature of 6500–7500 K. The purpose of installing a light source is to eliminate shadows in the image to improve the quality of the captured image. The background board is the most prominent black because black is most conducive to the subsequent segmentation of the potato image foreground and background.

### 2.3. Algorithm Flow Chart

**Figure 3:**
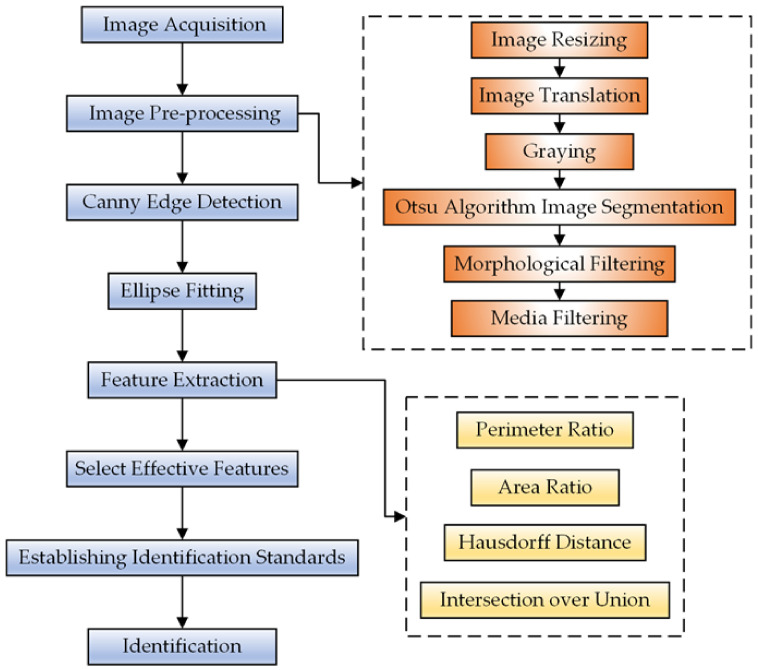
Flow chart of the irregular potato identification algorithm.

A flow chart identifying irregular potatoes is shown in Figure 3.

### 2.4. Image Preprocessing

The shape feature of the potato is not affected by factors such as lighting, color, and texture and is considered a stable feature [37]. To better extract the shape features of potatoes, the images need to be preprocessed. Because identifying irregular potatoes requires only contour information, the ultimate goal of image preprocessing is to retain the contour information of potatoes and eliminate information that is not relevant to the contour. Image preprocessing includes resizing, translation, graying, image segmentation, and filtering.

**Figure 4:**
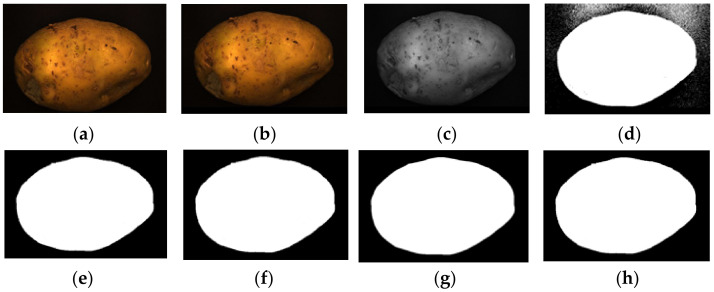
Image preprocessing results: (**a**) resizing, (**b**) translation, (**c**) graying, (**d**) binarization, (**e**) morphological filtering, (**f**) Gaussian filtering, (**g**) media filtering, and (**h**) bilateral filtering.

In the experiment, to improve the overall efficiency of the algorithm, the acquired potato image (3072 × 2048) is resized to 614 × 410 using region interpolation with a scaling factor of 0.2, as shown in Figure 4a. The results show that the efficiency of image processing is significantly improved after image scaling. As shown in Figure 4b, the potato is translated to the center of the image to clarify the potato’s contour and avoid extracting an incomplete contour. Graying is performed as shown in Figure 4b, and the weighted average method is often used, that is, the pixel values of B, G, and R channels are multiplied by different weights; the results are shown in Figure 4c. The redundant color information is eliminated after graying, which further improves the efficiency of image preprocessing. Then, the Otsu algorithm is used to identify the best threshold value for the foreground and background segments. Next, a binary image is obtained. Figure 4d shows the result after binarization by Otsu’s algorithm. A morphological filtering operation is performed to eliminate the noise points in the binary image; the results are shown in Figure 4e. After morphological filtering, all noise points in the binary image have been eliminated. However, the image contour becomes is not smooth after morphological filtering. Three methods of Gaussian filtering, median filtering, and bilateral filtering are used to smooth the edge contour. As shown in Figure 4f–h, Gaussian filtering and bilateral filtering do not have a good edge smoothing effect, instead blurring the image. Median filtering smooths the edge contour, achieving a positive result.

### 2.5. Edge Detection and Ellipse Fitting

**Figure 5:**
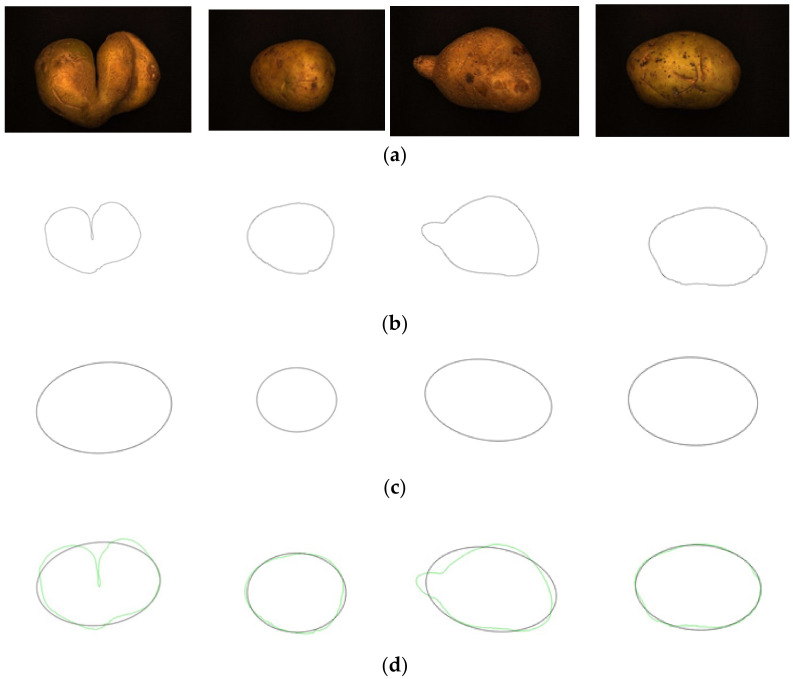
Contour images vs. ellipse images: (**a**) original images, (**b**) contour images, (**c**) ellipse images, and (**d**) overlay of profile images on ellipse images.

After obtaining the ideal binary image, canny edge detection is used to extract the potato image contours, as shown in Figure 5b. Canny edge detection can extract a complete and precise contour. A regular potato has a shape similar to an ellipse. In contrast, an irregular potato differs significantly from an ellipse. Ellipse fitting is important in target detection, target tracking, feature extraction, and image segmentation [38,39,40,41]. Therefore, the potato contour image is fitted to an ellipse, the contour image is compared with the fitted ellipse image, and the difference in shape between the two images is used to describe the degree of irregularity of the potato and thus identify irregular potatoes. Commonly used ellipse-fitting methods include least squares, Hough transform, and edge tracing [42]. The ellipse-fitting method based on Hough transformation is insensitive to isolated points and can solve cases in which the ellipse is occluded. However, because it considers each edge pixel point of the image for voting, the computation complexity is significantly increased, requiring increased memory resources [43], somewhat limiting its application. Compared to the Hough transform approach, the edge-tracking method is more efficient in terms of computation and storage. Moreover, it utilizes the information of geometric correlation between points [44]. The least-squares method is widely used because of its ease of implementation and its ability to obtain closure results [45]. However, the least-squares process is sensitive to noise points, which must be strictly controlled. Considering the real-time practicality of the algorithm, the least-squares method is proposed for use in ellipse fitting of potato contours.

The potato contour is fitted to an ellipse using a least-squares ellipse-fitting method, as shown in Figure 5c,d. The difference between the potato contour image and the fitted ellipse image shows that the regular potato contour and the fitted ellipse approximately overlap. However, there is a significant difference between the contour of the irregular potato and the fitted ellipse. Therefore, the similarity between the contour and fitted ellipse images can be used to identify irregular potatoes.

### 2.6. Feature Extraction

Three feature parameters are proposed to characterize the degree of potato irregularity based on ellipse fitting: the ratio between the potato contour perimeter and the fitted ellipse perimeter, the ratio between the potato contour area and the fitted ellipse area, and the Hausdorff distance between the potato contour image and the fitted ellipse image.

#### 2.6.1. Perimeter Ratio

Regular potatoes have a shape similar to an ellipse, whereas irregular potatoes are primarily irregular in shape and differ considerably from an ellipse. Therefore, the potato contour perimeter and the fitted ellipse perimeter ratio are characteristic parameters used to characterize the degree of potato irregularity. C is the perimeter ratio, as shown in Equation (1).

(1) $$C=\frac{C_{p}}{C_{e}}$$

where C_p_ is the potato contour perimeter, and C_e_ is the fitted ellipse perimeter.

#### 2.6.2. Area Ratio

Similarly, the ratio of the area of the potato contour area to the fitted ellipse area is used as a characteristic parameter to characterize the degree of potato irregularity. A is the ratio of area, as shown in Equation (2).

(2) $$A=\frac{A_{p}}{A_{e}}$$

where A_p_ is the potato contour area, and A_e_ is the fitted ellipse area.

#### 2.6.3. Hausdorff Distance

Suppose that the fitted elliptical coordinate points are p(x_k_, y_k_), where k = 1, 2, N; N is the number of ellipse contour points; and x_k_ and y_k_ are the coordinates of the fitted ellipse points in the image. The potato contour coordinates are q(u_k_, v_k_), where k = 1, 2, n; n is the number of contour points; and u_k_ and v_k_ are the coordinates of potato contour points in the image. Assume that the ellipse points fitted by the least-squares method constitute set P, and the potato contour points constitute set Q. Because the shape of the regular potato image is similar to an ellipse and the form of the irregular potato is significantly different from an ellipse, the feature extraction of the irregular potato is transformed into a measure of the similarity between sets P and Q. To describe the similarity between sets P and Q, traditional Euclidean distance is used to measure the similarity between sets. It does not consider the relative positions of the points between the two sets nor the overall characteristics of the sets. However, the Hausdorff distance integrates the relative positions of object and complete shapes to calculate the similarity. Hausdorff distance has been an indispensable tool for solving computer vision and pattern recognition problems [46]. Therefore, Hausdorff distance is used as the irregularity feature parameter of potatoes.

Considering the contour of the target shape as an unordered set of points, suppose that the fitted ellipse image constitutes set P, and the potato boundary contour image constitutes set Q.

$$\begin{array}{l} P=\left\{p_{1},p_{2},\ldots ,p_{n}\right\}\\ Q=\left\{q_{1},q_{2},\ldots ,q_{n}\right\} \end{array}$$

**Figure 6:**
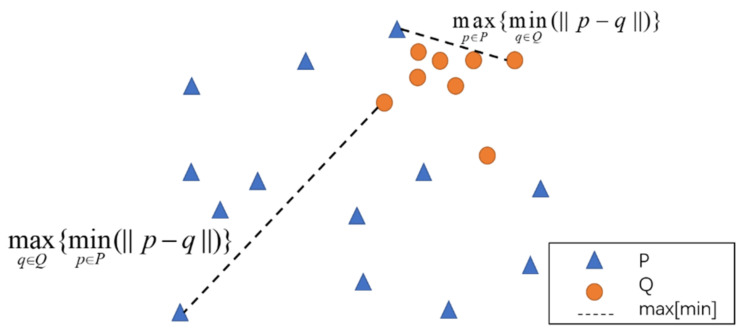
Diagram of Hausdorff distance calculation.

Then, the Hausdorff distance between sets P and Q is defined as:

(3) H(P,Q)=max(h(P,Q),h(Q,P))

where H(P, Q) represents the bidirectional Hausdorff distance between sets P and Q; and h(P, Q) and h(Q, P) are the unidirectional Hausdorff distances from set P to set Q and from set Q to set P, respectively. The mathematical expressions are as follows:

(4) $$\begin{array}{l} h(P,Q)=\max_{p\in P}\{\min_{q\in Q}(||p\text{-}q||)\}\\ h(Q,P)=\max_{q\in Q}\{\min_{p\in P}(||p\text{-}q||)\} \end{array}$$

where ∥•∥ represents the L2 norm. Calculate the distances between all points in sets P and Q; then, select the point farthest from set Q and calculate the distance between P and its nearest neighbor in Q. Take the value of this distance as h(P, Q); similarly, calculate h(Q, P), as shown in Figure 6.

The bidirectional Hausdorff distance between P and Q takes the maximum of h(P, Q) and h(Q, P), and the bidirectional Hausdorff distance measures the maximum non-matching degree between sets P and Q. The shorter the Hausdorff distance between two sets, the more similar they are.

#### 2.6.4. Intersection over Union

Intersection over Union (IoU) is widely used in deep learning and measures the degree of overlap between the prediction box and the ground truth box in object detection. The formula for calculating IoU is shown in Equation (5).

(5) $$\mathrm{IoU}=\frac{\;\mathrm{Area}\;\mathrm{of}\;\mathrm{Overlap}}{\mathrm{Area}\;\mathrm{of}\;\mathrm{Union}}$$

**Figure 7:**
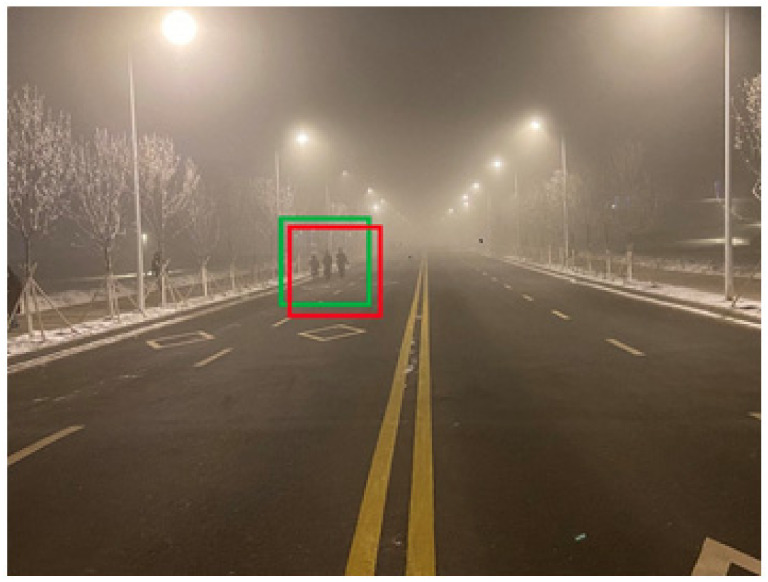
Object detection. The green box represents the ground truth box and the red box represents the prediction box.

As shown in Figure 7, the prediction box (red) does not overlap with the ground truth box (green) when detecting road pedestrians. To evaluate the performance of the object detection algorithm, IoU is used to represent the degree of overlap between the prediction box and the ground truth box.

If IoU is equal to 1, the prediction box completely overlaps with the truth box, and the algorithm performs exceptionally well. On the contrary, if IoU is equal to 0, the prediction box does not intersect with the truth box, and the algorithm fails. In other words, the degree of overlap between the prediction box and the truth box reflects the excellent or inadequate performance of the algorithm.

In this paper, if we consider the potato contour as the truth box and the fitted ellipse as the prediction box, then we get Equation (6).

(6) $$\mathrm{IoU}=\frac{\mathrm{ContourArea}\cap EllipseArea}{\mathrm{ContourArea}\cup EllipseArea}$$

As shown in Figure 5d, the potato contour and the fitted ellipse must have intersections. The intersection and union of regular potatoes are relatively close, whereas the intersection and union of irregular potatoes show apparent differences. Therefore, IoU is used as a characteristic parameter to identify irregular potatoes; the farther the IoU is from 1, the more likely it is to be an irregular potato.

## 3. Results

### 3.1. Characterization Ability of Features

#### 3.1.1. Perimeter Ratio and Area Ratio

To verify the characterization ability of perimeter ratio and area ratio in identifying irregular potatoes, 25 irregular potatoes and 50 regular potatoes are deliberately selected from the sample to form an example set for analysis of perimeter ratio and area ratio characterization ability. The deliberate selection is intended to make the pieces as diverse as possible to include both irregular and regular potatoes and ensure a broad sample set, after which the perimeter and area ratio is calculated separately for each sample.

**Figure 8:**
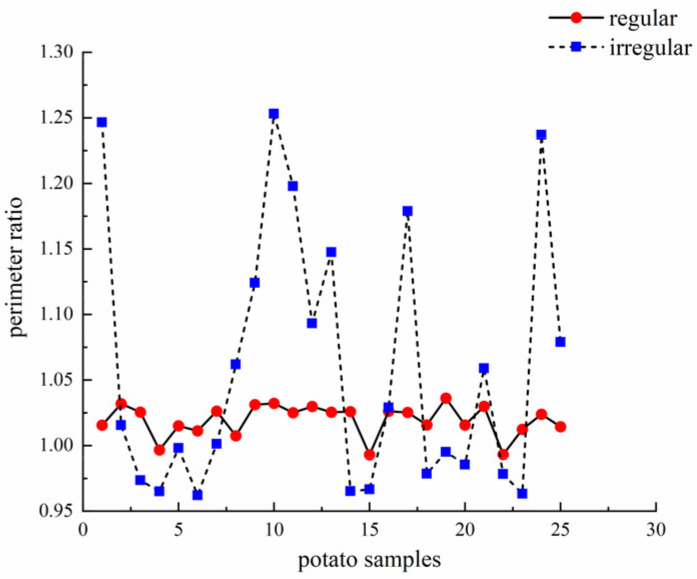
Perimeter ratio of potatoes.

The ratios of contour perimeter and ellipse perimeter of irregular and regular potatoes are shown in Figure 8 (only 25 regular potatoes are shown). The perimeter ratios of regular potatoes are both close to 1 and more stable compared to irregular potatoes. However, there is no clear boundary between the perimeter ratios of irregular and regular potatoes due to the extreme instability of the perimeter ratios of irregular potatoes. Furthermore, some irregular potatoes have perimeter ratios closer to 1 than regular potatoes, as in samples 2, 5, and 7. Therefore, finding an actual threshold to distinguish between irregular and regular potatoes is impossible.

**Figure 9:**
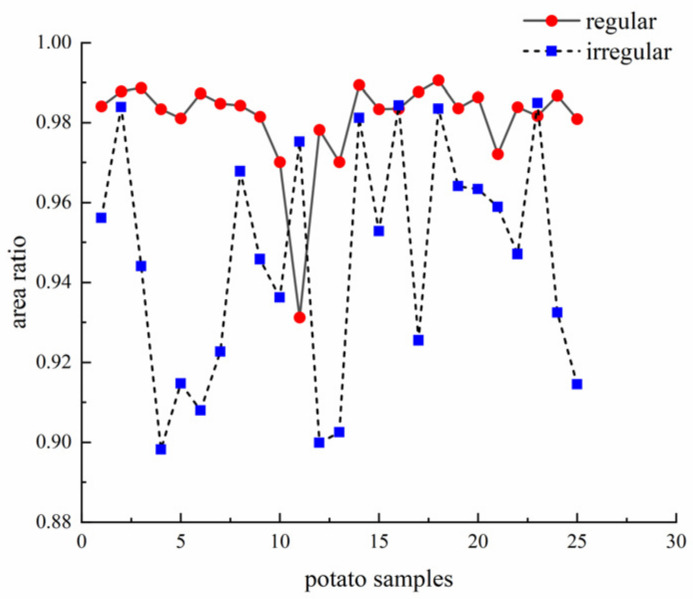
Area ratio of potatoes.

The ratios of contour area and ellipse area for irregular and regular potatoes are shown in Figure 9. Similar to the perimeter ratio, the area ratios of the regular potatoes are all close to 1. However, a definite threshold could not be found to distinguish irregular from regular potatoes.

#### 3.1.2. Hausdorff Distance

**Figure 10:**
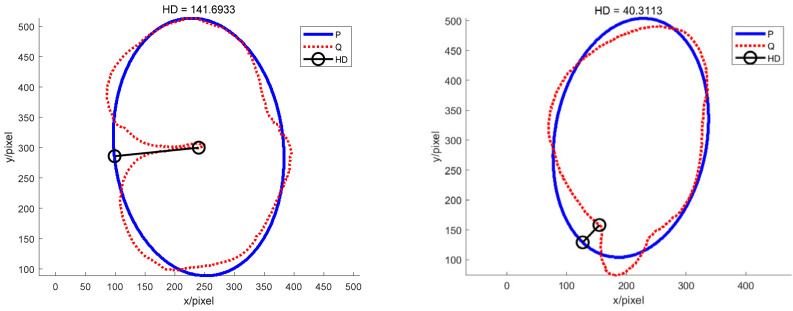
Hausdorff distance calculation results for irregular potatoes.

**Figure 11:**
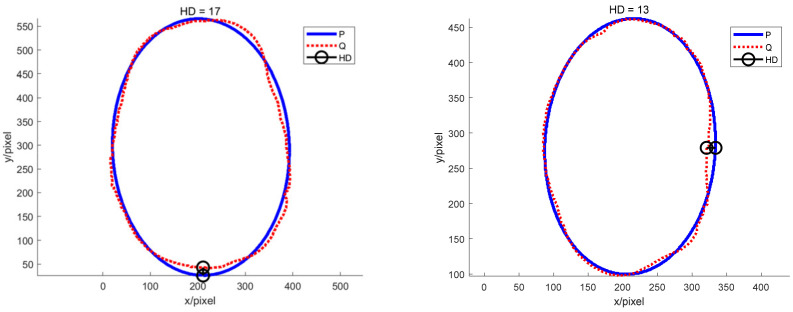
Hausdorff distance calculation results for regular potatoes.

Hausdorff distance is used as a feature parameter of irregular potatoes to measure the matching degree between the potato contour image and the fitted ellipse image. The larger the value of Hausdorff distance, the higher the degree of non-match between the two images. The results of the Hausdorff distance calculation for irregular potatoes are shown in Figure 10, and the results of the Hausdorff distance calculation for regular potatoes are shown in Figure 11.

The P set in Figure 10 and Figure 11 represents the fitted ellipse, set Q represents the potato contour, and HD represents the Hausdorff distance between sets P and Q. The Hausdorff distances for the two potatoes in Figure 10 are 141 and 40, respectively, and in Figure 11, the Hausdorff distances are 17 and 13. The calculation results showed that the Hausdorff distance value of irregular potatoes is significantly larger than that of regular potatoes. The larger the Hausdorff distance value of irregular potatoes, the higher the degree of non-match between the two sets and the higher the probability that the potatoes are irregular.

**Figure 12:**
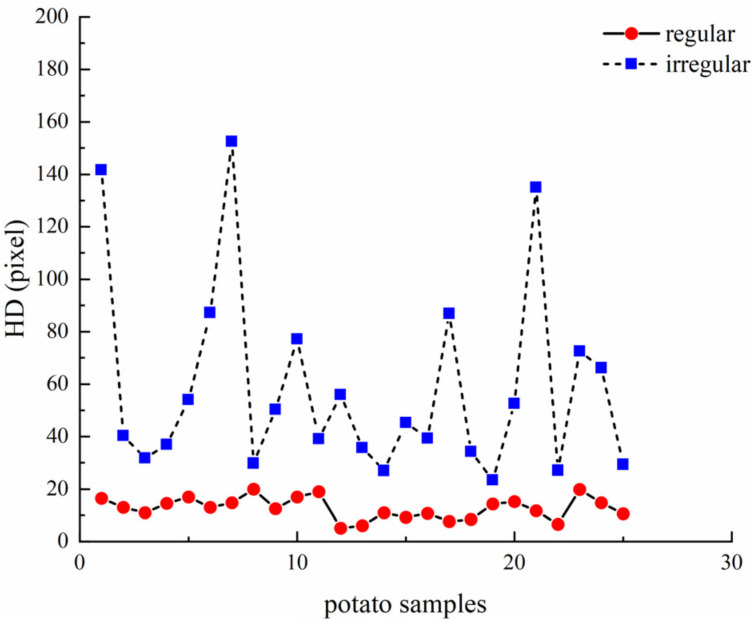
Hausdorff distance values for irregular and regular potatoes.

To further analyze the characterization ability of Hausdorff distance in identifying irregular potatoes, 25 different irregular potatoes and 50 regular potatoes are intentionally selected from the sample to form an example set for analysis of Hausdorff distance characterization ability. The purpose of deliberate selection is to make the piece as diverse as possible by including a wide variety of irregular and regular potatoes and ensure a broad sample set. Figure 12 represents the Hausdorff distance distribution of the samples. The Hausdorff distance values for the irregular and regular potatoes have clear thresholds.

As shown in Table 2, the minimum value of Hausdorff distance for irregular potatoes is 23.43, whereas the maximum value of Hausdorff distance for regular potatoes is 20. There is no intersection of Hausdorff distance values for irregular and regular potatoes, with clear thresholds. The mean and standard deviation show that the Hausdorff distances of irregular potatoes are generally large and fluctuate significantly, which also verifies the complexity and variability of irregular potato shapes from the side. Therefore, the Hausdorff distance values of irregular and regular potatoes are vastly different, and the Hausdorff distance can effectively characterize whether a potato is irregular or regular.

#### 3.1.3. IoU

Using IoU to identify irregular potatoes, the closer the value of IoU is to 1, the more likely it is to be a regular potato; the inverse is true for irregular potatoes. Table 3 represents the IoU calculation results for regular and irregular potatoes. IoU values of regular potatoes are more significant than those of irregular potatoes because the shape of the regular potato contour is closer to an ellipse.

**Figure 13:**
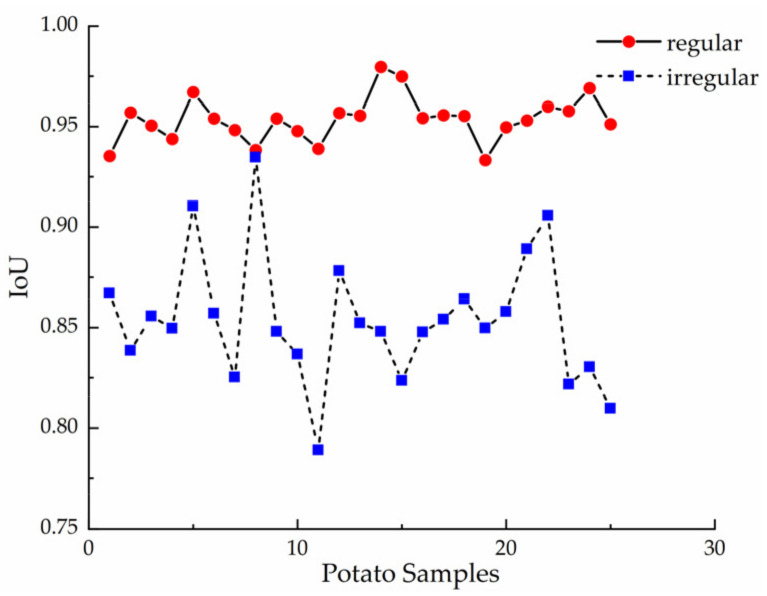
IoU values for regular and irregular potatoes.

Similarly, 25 irregular potatoes and 50 regular potatoes are selected from the samples to constitute the sample set for analysis of IoU characterization ability. The calculated IoU values for each piece are shown in Figure 13.

As shown in Table 4, the IoU values of the regular potatoes are highly stable, all greater than 0.93. The majority of the IoU values for irregular potatoes are below 0.9. The maximum value is 0.9347, which is very close to the value for regular potatoes.

### 3.2. Establishing Identification Standards for Irregular Potatoes

In summary, the two characteristic parameters, perimeter ratio and area ratio, are less well characterized than the Hausdorff distance and the IoU. Therefore, Hausdorff distance and IoU are used as characteristic parameters to describe irregular potatoes. Based on the previous analysis, we can conclude the identification standards of irregular potatoes, as shown in Table 5.

### 3.3. Evaluation Metrics for Recognition Rate

Many recognition rate evaluation metrics for algorithms are available, and a single evaluation metric is not comprehensive enough in the practical application process. Therefore, in this study, we use four metrics to evaluate the algorithm’s accuracy: accuracy, precision, recall, and F1 score.

Accuracy is defined as the number of correctly classified samples as a percentage of all models. Accuracy is calculated as shown in Equation (7). The specific definitions of TP, TN, FP, and FN are shown in Table 6.

(7) $$\mathrm{Accuracy}=\frac{\mathrm{TP}+\mathrm{TN}}{\mathrm{TP}+\mathrm{FP}+\mathrm{TN}+\mathrm{FN}}$$

Precision is defined as the proportion of samples with a predicted value of 1 and an actual value of 1 among all models with a predicted value of 1. Precision is calculated as shown in Equation (8).

(8) $$\mathrm{Precision}=\frac{\mathrm{TP}}{\mathrm{TP}+\mathrm{FP}}$$

Recall is defined as the proportion of samples with a predicted value of 1 and an actual value of 1 among all models with a true value of 1. Recall is calculated as shown in Equation (9).

(9) $$\mathrm{Recall}=\frac{\mathrm{TP}}{\mathrm{TP}+\mathrm{FN}}$$

F1 score is a statistical metric used to evaluate the recognition rate of a binary classification model. It combines the precision and recall of the classification model at a given time. The F1 score can be considered a weighted average of the model precision and recall, with a maximum value of 1 and a minimum value of 0. Larger values indicate better models. The F1 score is calculated as shown in Equation (10).

(10) $$F1\;\mathrm{score}=2\cdot \frac{\;\mathrm{Precision}\cdot \mathrm{Recall}}{\;\mathrm{Precision}+\mathrm{Recall}}$$

The number of positive and negative samples in the experiment is not balanced; therefore, it is not appropriate to use the accuracy metric to evaluate the algorithm. In this paper, we estimate the algorithm’s performance using a combination of precision, recall, and F1 scores.

### 3.4. Experimental Validation

From the irregular potato identification standard, 98 normal potatoes and 50 irregular potatoes are randomly selected as the validation set to identify irregular potatoes. The identification results for Hausdorff distance and IoU are shown in Table 7 and Table 8, respectively.

A comparison of Table 7 and Table 8 shows that Hausdorff distance is prone to incorrectly identifying regular potatoes as irregular potatoes, whereas no incorrect identification occurs when IoU is used to identify regular potatoes. However, the Hausdorff distance has a more vital characterization ability in identifying irregular potatoes, with fewer incorrect identifications.

## 4. Discussion

### 4.1. Shooting Perspective

**Figure 14:**
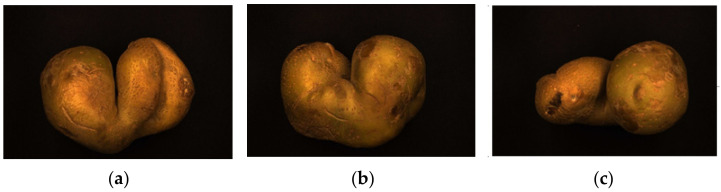
Images of an irregular potato from different shooting perspectives: (**a**) front shot, (**b**) back shot, and (**c**) side shot.

The perspective plays a vital role in the performance of the captured 2D potato images and the proposed method. For irregular potatoes, the shape is complex and variable, and the morphological characteristics of potatoes differ depending on the shooting angle. Figure 14 shows three views of an irregular potato with differing appearance characteristics.

**Figure 15:**
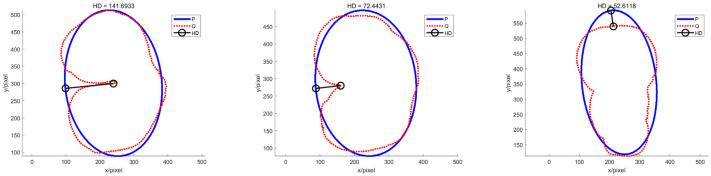
Hausdorff distance values of irregular potatoes from different perspectives.

Because the morphological characteristics of potatoes differ according to the shooting perspective, the extracted contours and fitted ellipses, as well as the Hausdorff distance values, also differ according to the shooting perspective. Therefore, the Hausdorff distance values of an irregular potato from each of the three perspectives are calculated as shown in Figure 15.

As shown in Figure 15, the Hausdorff distance values vary depending on the shooting angle. However, the change in Hausdorff distance values does not affect the final identification of the potatoes, as all three Hausdorff distance values are more significant than the discrimination threshold for irregular potatoes.

Table 9 shows the IoU values of irregular potatoes for the three viewpoints, similar to the Hausdorff distance. However, the different views have different IoU values, which does not influence the final recognition results.

**Figure 16:**
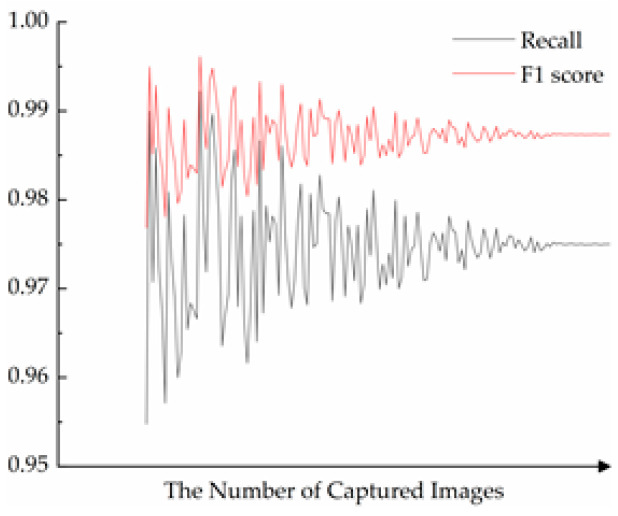
The influence of shooting perspective on algorithm robustness.

In the experiment, the lens of the industrial camera faces downward vertically, and the bottom of the potato cannot be photographed. Assuming that the camera angle is fixed, the potato is placed in a position in which it can stand naturally. If there happens to be a natural standing position in which the irregular part of the potato is entirely at the bottom of, the otherwise irregular potato is photographed as a regular potato. Once such a shooting perspective exists, irregular potatoes can be incorrectly treated as regular potatoes, directly affecting the final recognition rate. Therefore, at the two-dimensional level, the perspective of the shot is crucial. Figure 16 shows the influence of the shooting angle on the algorithm’s robustness.

As shown in Figure 16, the recall and the F1 scores fluctuate considerably depending on the shooting angle in the case of a small sample size. As the sample size increases, the recall and the F1 scores fluctuate less and gradually converge to a specific value. Potato images can be acquired from multiple angles to avoid false recognition due to view angle. The final Hausdorff distance is taken as the maximum value under multiple view angles. The IoU is taken as the minimum value.

### 4.2. Datasets and Thresholds

The existing public potato dataset has the disadvantages of cluttered shooting background, low image quality, and lack of irregular potato samples. Therefore, in this study, we use a self-built visual acquisition system to capture potato images. To ensure that the dataset is representative, potato samples are selected by six experienced potato graders. Groups of two workers are divided into three groups. The first group specializes in selecting potatoes with minor irregularities, and the second group specializes in selecting potatoes with severe irregularities. After the first two groups are completed, a third group categorizes irregular potatoes as slightly or severely irregular based on the results of the first two groups. Finally, the six workers jointly select the samples of regular potatoes, forming the dataset used in this study.

The size of the original dataset image is 3072 × 2048. To reduce the CPU computation requirements and improve the running efficiency of the algorithm, the image is scaled to 614 × 410. Although the change in the image resolution compromises some texture information, the shape of the potato does not change as a result of image scaling, and the texture information does not affect the shape of the discriminated potato. The Hausdorff distance threshold (21) is derived based on an image size of 614 × 410. Therefore, the threshold is valid as long as the image size is guaranteed to be 614 × 410. The image resolution does not influence the threshold for identifying irregular potatoes using IoU. Admittedly, the threshold is not constant and fluctuates depending on the potato variety.

### 4.3. Advantages of the Algorithm

Deep learning methods require large datasets to complete model training. Unfortunately, for irregular potato identification, a large and diverse dataset has not been compiled for use by researchers [47]. Moreover, labeling training data is a time-consuming and labor-intensive task [48]. Furthermore, the model training process is associated with demand for CPU and GPU computing power; the less computing power, the longer required for training [49,50]. In addition, the model’s design is crucial, and a flawed model is likely to overfit or underfit [51]. In this study, we use traditional image processing methods to identify irregular potatoes by combining the above factors.

Currently, many parameters describe shapes, but they are generally divided into region-based feature parameters and boundary-based feature parameters. In traditional image processing methods, geometric features, Fourier descriptors, and invariant moments are usually used to extract potato shape features. Invariant moments are region-based feature parameters. Geometric features and Fourier shape descriptors are boundary-based feature parameters [52]. In this paper, we propose, for the first time, the use of Hausdorff distance and IoU based on ellipse fitting to describe the shape features of potatoes. With the dataset built in this paper, the geometric features, Fourier descriptors, invariant moments, Hausdorff distance, and IoU are used to identify irregular potatoes. The geometric feature method first calculates the smallest external rectangle of the potato view. It then uses the ratio of the area of the smallest external rectangle and the contour as a feature parameter to identify irregular potatoes. The Fourier descriptors take the Fourier transform of the potato boundary information as the shape feature, transform the contour feature from the spatial domain to the frequency domain, and extract the frequency domain information as the feature vector of the image. Generally, the first 10–15 dimensions of the feature vector are taken; in this paper, the first 15 dimensions of the feature vector are taken to describe the shape of potatoes. We use BP neural networks to implement the classification. The invariant moment method extracts 10 invariant moments in the image after edge detection to represent the shape of potatoes. Then, the calculated invariant moments feature parameters are input to the SVM to achieve the classification. The recognition rate and execution time of each feature are shown in Table 10.

As shown in Table 10, the Hausdorff distance and the IoU achieve excellent performance, with the highest recognition rate and the shortest execution time. The reasons for such results are as follows.

1. The shape of irregular potatoes is complex, and the perimeter and area ratios take into account global features but cannot take into account local features;
2. Fourier descriptors are effective for round and oval potatoes but easily make incorrect predictions for irregular potatoes with irregular contours;
3. Invariant moments have a good recognition rate, but to obtain higher recognition accuracy, the feature vectors of the three classical shape features are often multidimensional. As a result, machine learning classifiers, such as BP neural networks and SVM, are required to identify irregular potatoes, increasing the identification time.

In this paper, a method of irregular potato identification based on Hausdorff distance and IoU is proposed, which does not require massive datasets, data training, and high computing power, instead requiring comparatively affordable hardware equipment. The proposed method recognizes only simple threshold segmentation, significantly reducing the complexity of the algorithm. More importantly, the algorithm achieves excellent performance, with a maximum F1 score of 0.9796.

## 5. Conclusions

We propose a new algorithm to identify irregular potatoes, achieving excellent performance, with a maximum F1 score of 0.9796, making it capable of meeting practical industrial needs. The potato contour is extracted by canny edge detection, and the contour is fitted to an ellipse using the least-squares method. Four feature descriptors, perimeter ratio, area ratio, Hausdorff distance, and IoU are proposed based on ellipse fitting. The experimental results show that the characterization ability of perimeter ratio and area ratio is inferior to that of Hausdorff distance and IoU. The proposed algorithm uses Hausdorff distance and IoU separately to identify irregular potatoes, significantly reducing the dimensionality of feature parameters and the computational complexity of the algorithm. More importantly, the algorithm proposed in this paper does not require a training process and can accurately identify irregular potatoes using only simple threshold segmentation. The algorithm proposed of this paper provides a theoretical basis and technical reference for detection of irregular potatoes. It can also be extended to detection of other irregular agricultural products, which is essential for promoting the appreciation and processing of farm products.

To ensure the robustness of the algorithm, the following factors are critical:

1. Potato shooting perspective;
2. A clear and complete contour of the captured potato images.

The potato image dataset was obtained under good lighting conditions in a laboratory. In the future, it will be a challenging task to acquire potato images with more complex lighting environments and backgrounds in order to build datasets with larger samples. In addition, potatoes present with a wide variety of surface defects. Therefore, exploration of efficient identification algorithms to detect a wider range of defects with the help of advanced computer vision technology will constitute a principal research task in the future.

## Figures and Tables

**Table 1:** Principal parameters of the vision acquisition system.

Parameter	Value
Camera model	HIKROBOT-MV-CA060-10GC
Country	China
Camera working distance	300 mm
Lens focal	12 mm
Field of view size	300 mm × 200 mm
Sensor type	CMOS
Resolution	3072 × 2048
Pixel size	2.4 μm
Frame rate	17 fps
Light source Color temperature	LED strip light source 6500–7500 K

**Table 2:** Maximum, minimum, mean, and standard deviation of Hausdorff distance.

Parameter	Irregular Potatoes	Regular Potatoes
Maximum	152.46	20.00
Minimum	23.43	5.09
Mean	58.86	12.72
Standard deviation	36.72	4.30

**Table 3:** IoU calculation results for regular and irregular potatoes.

Type	Number	Contour	Ellipse	Intersection	Union	IoU
Regular	1	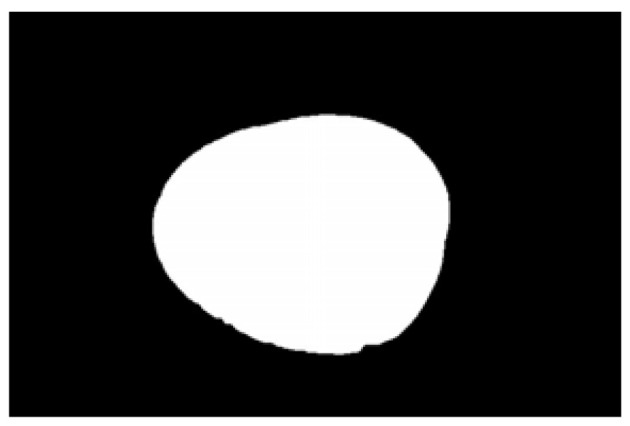	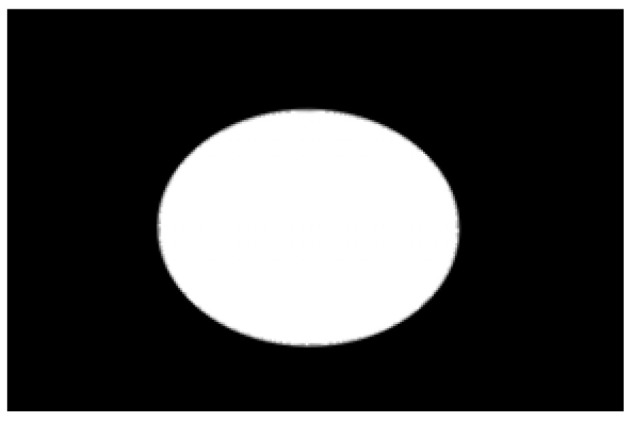	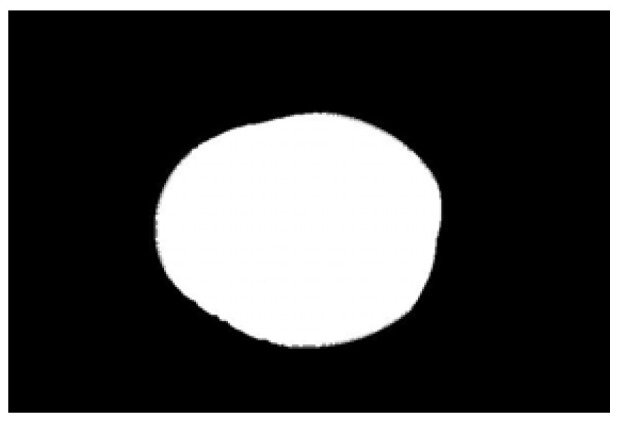	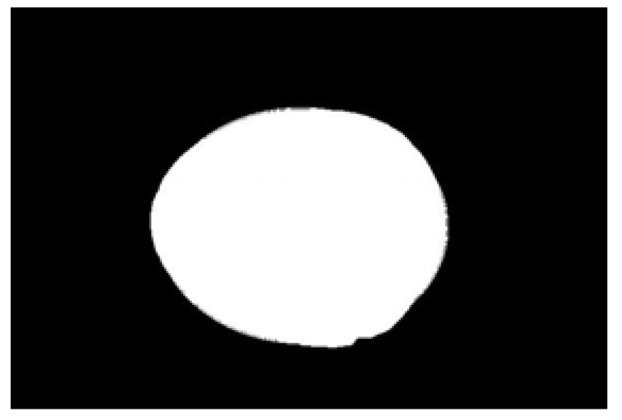	0.9353
	2	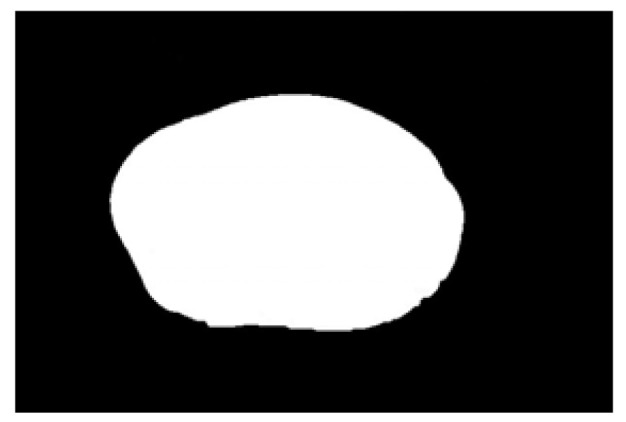	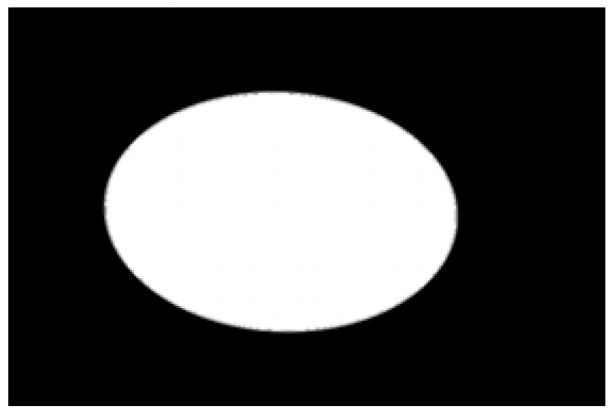	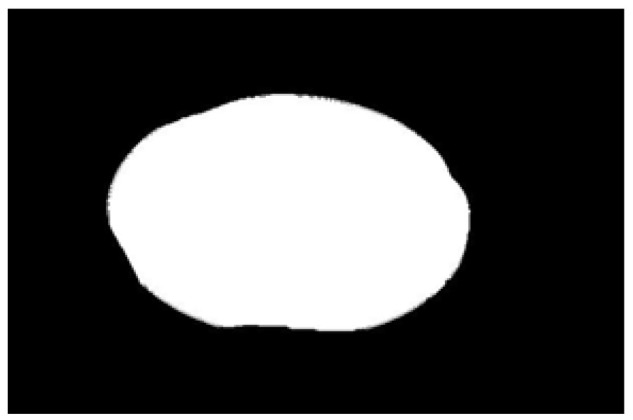	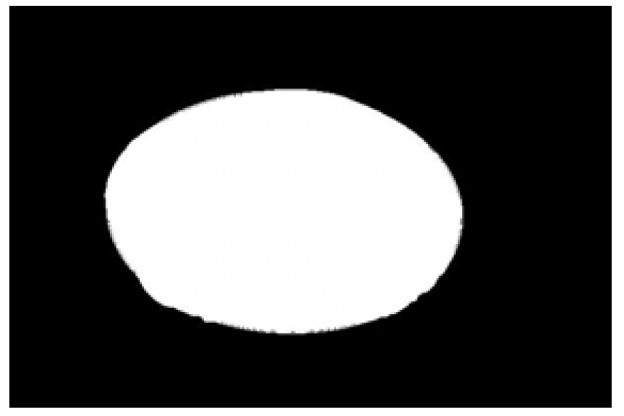	0.957
Irregular	3	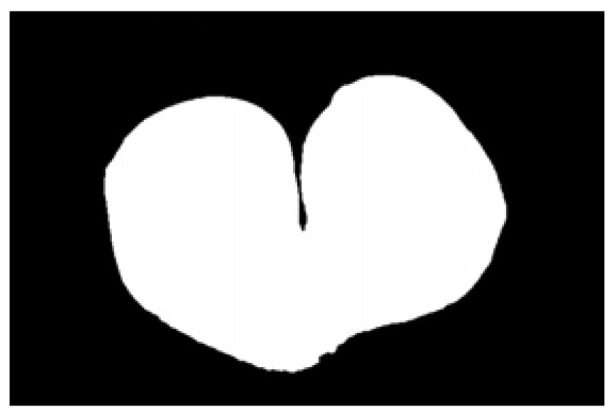	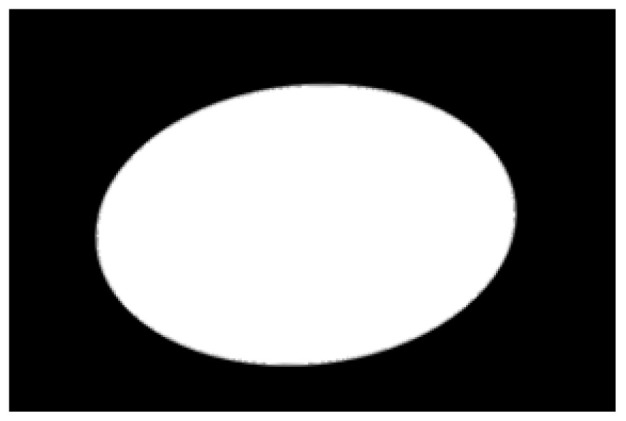	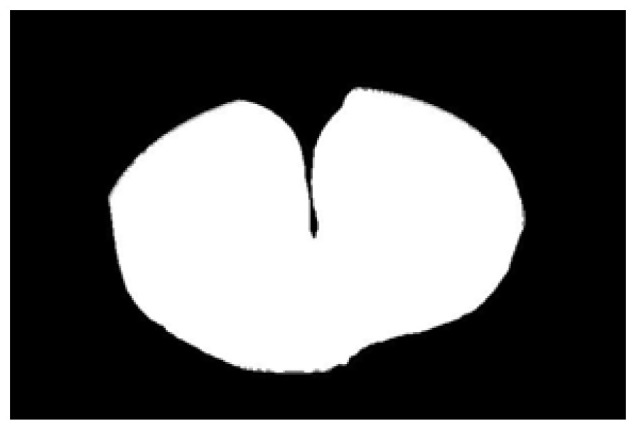	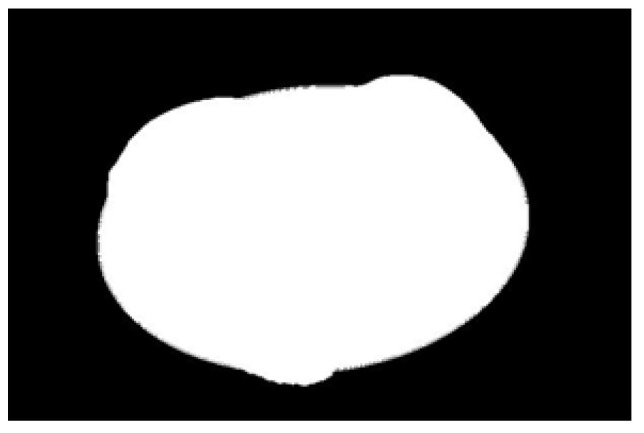	0.867
	4	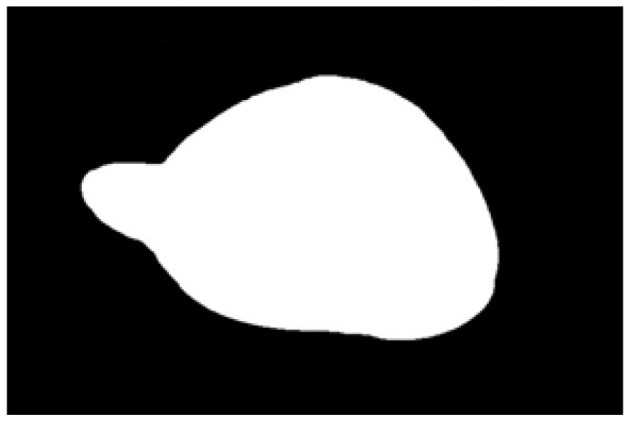	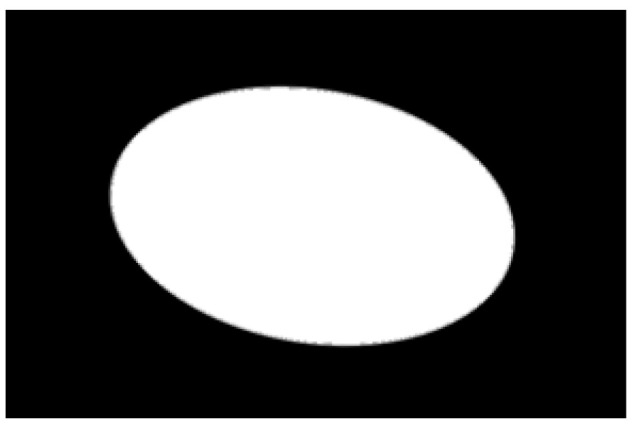	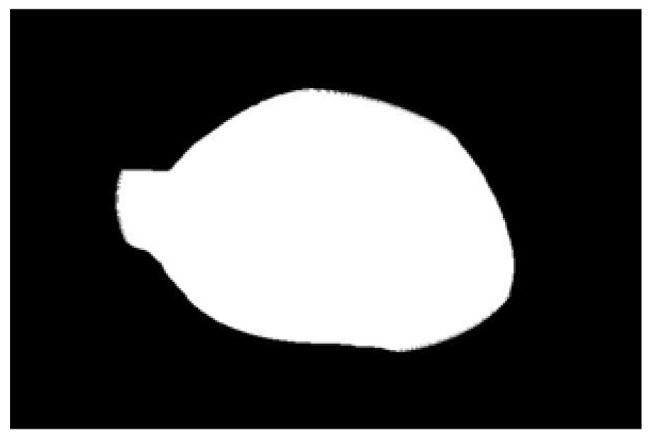	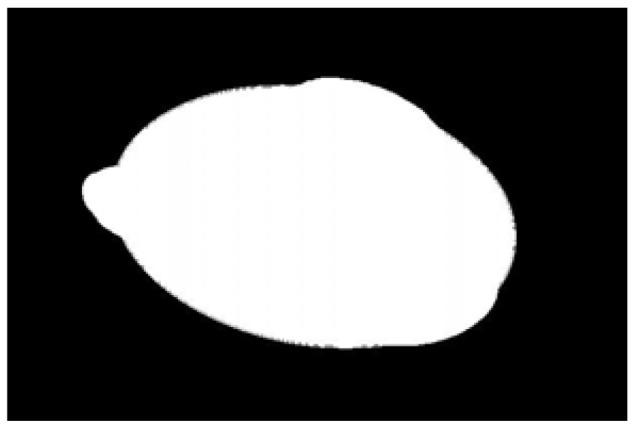	0.8496

**Table 4:** Maximum, minimum, mean, and standard deviation of IoU.

Parameter	Irregular Potatoes	Regular Potatoes
Maximum	0.9347	0.9796
Minimum	0.7890	0.9333
Mean	0.8538	0.9536
Standard deviation	0.0321	0.0112

**Table 5:** Irregular potato identification threshold.

Feature	Regular Potatoes	Irregular Potatoes
Hausdorff Distance	Less than or equal to 21	Greater than 21
IoU	Greater than 0.925	Less than or equal to 0.925

**Table 6:** Specific definitions of TP, TN, FP, and FN.

	P (Positive, 1)	N (Negative, 0)
T (True, 1)	TP (Positive samples predicted by the model to be positive)	TN (Negative samples predicted by the model to be negative)
F (False, 0)	FP (Negative samples predicted by the model to be positive)	FN (Positive samples predicted by the model to be negative)

**Table 7:** Identification results using Hausdorff distance.

	True Irregular	True Regular
Identified Irregular	49 (TP)	3 (FP)
Identified Regular	1 (FN)	95 (TN)

**Table 8:** Identification results using IoU.

	True Irregular	True Regular
Identified Irregular	48 (TP)	0 (FP)
Identified Regular	2 (FN)	98 (TN)

**Table 9:** IoU calculation results for irregular potatoes with multiple views.

Perspective	Contour	Ellipse	Intersection	Union	IoU
1	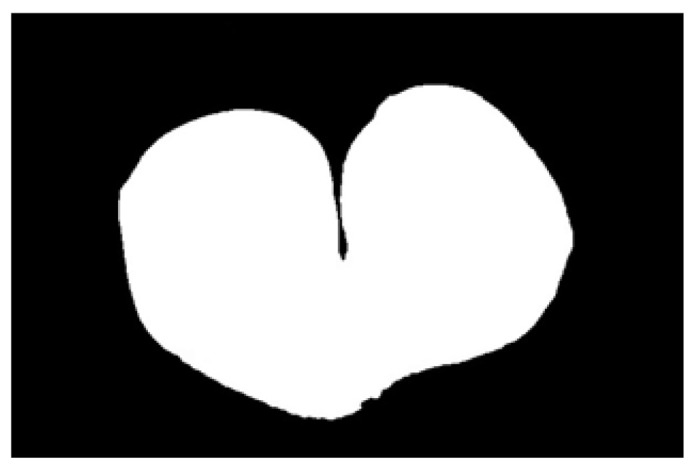	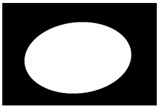	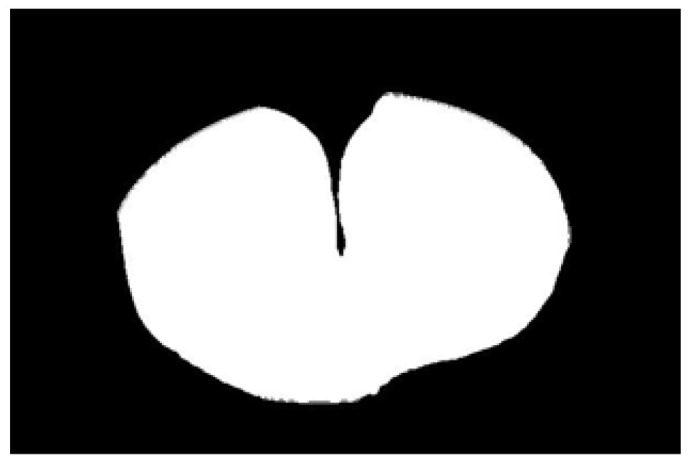	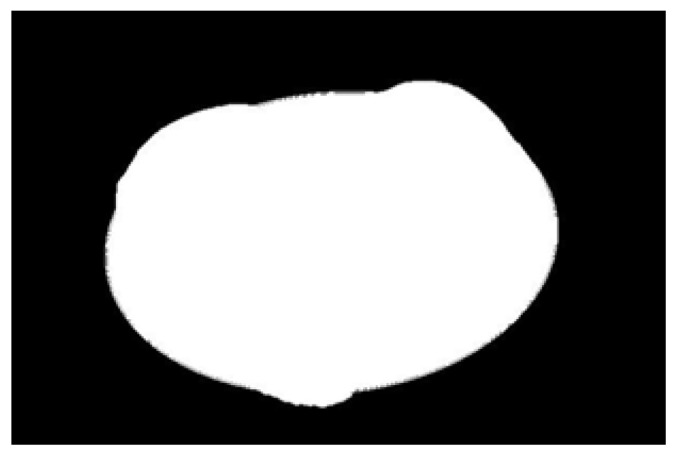	0.867
2	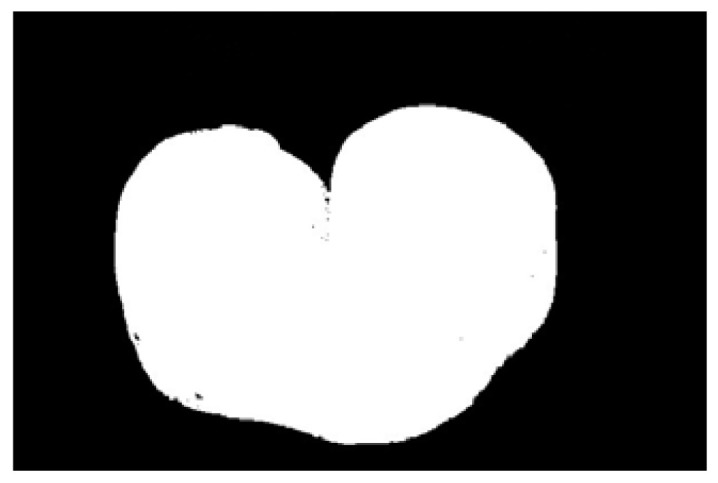	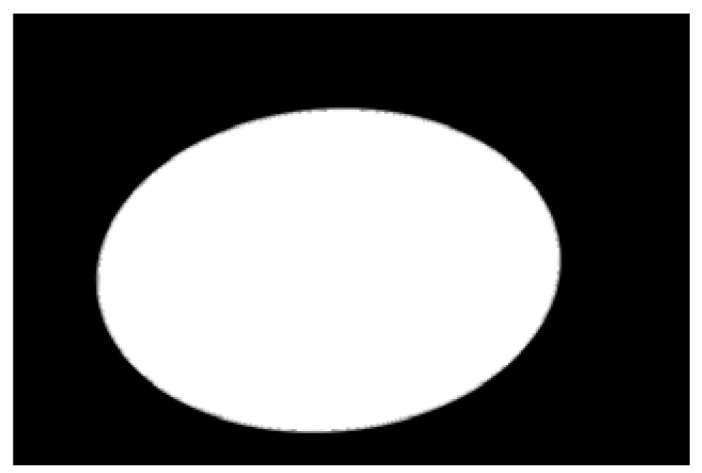	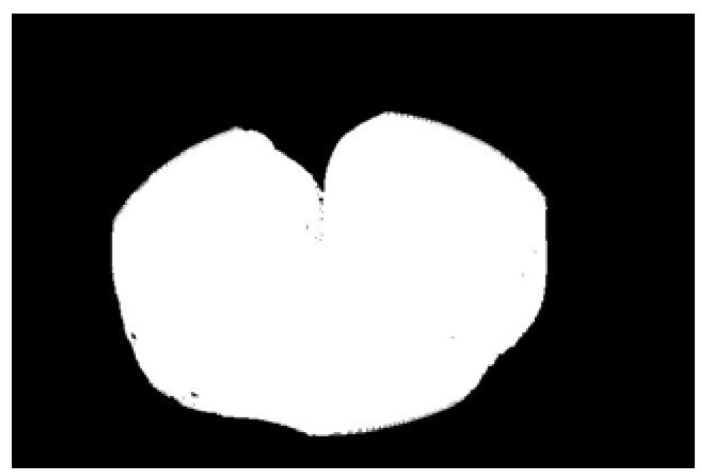	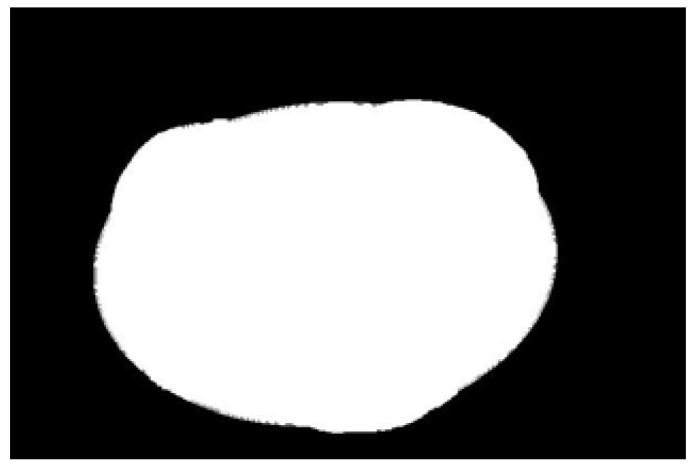	0.8668
3	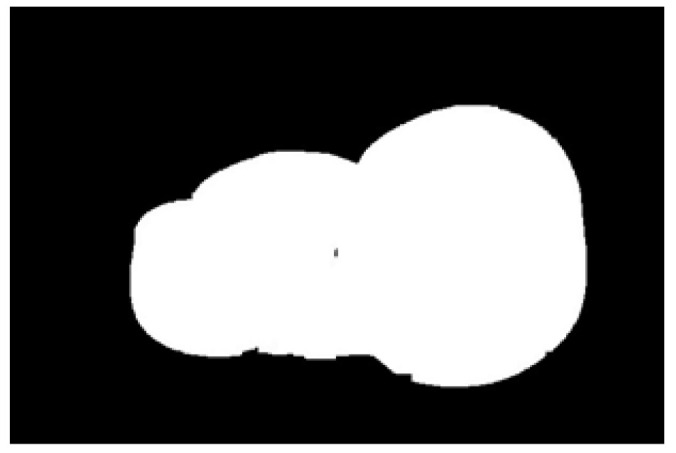	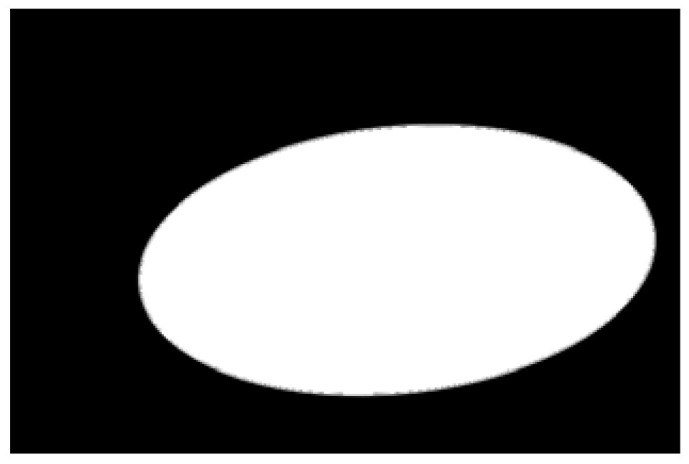	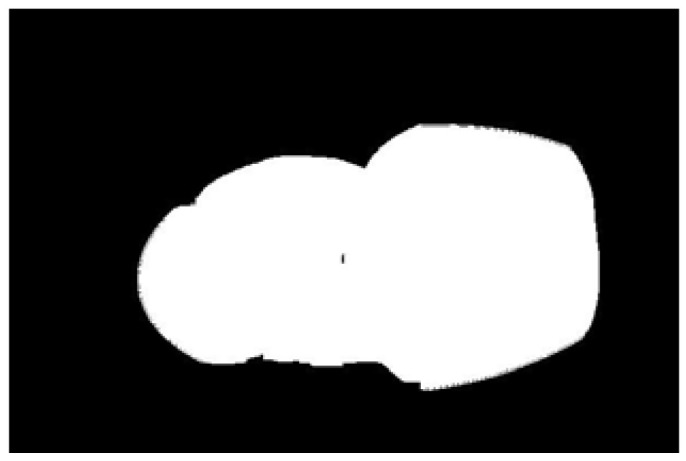	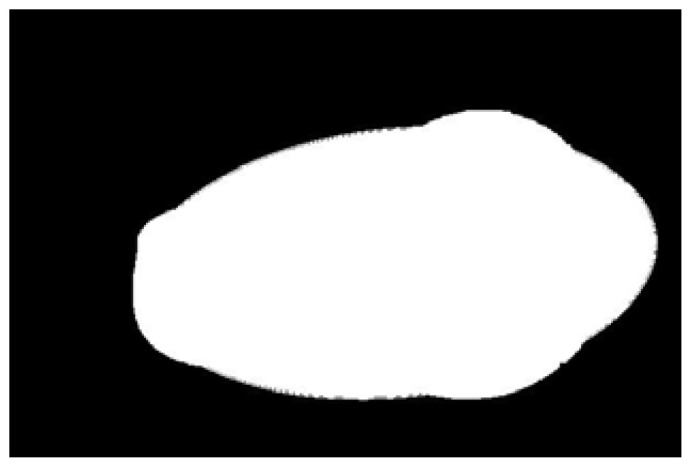	0.8185

**Table 10:** Recognition rate and execution time with different features.

Feature	Precision	Recall	F1 Score	Executive Time (ms)
Geometric Features	0.86	0.8113	0.8349	98
Fourier Descriptors	0.96	0.8276	0.8889	296
Invariant Moments	0.92	0.9019	0.9109	245
Hausdorff Distance	0.9423	0.98	0.9608	86
IoU	1	0.96	0.9796	79

## Data Availability

Data are not publicly available due to privacy considerations.

## Appendix A

The seven major types of potatoes:
